# Association between the ferritin level and risk of gestational diabetes mellitus: A prospective cohort study

**DOI:** 10.1371/journal.pone.0322719

**Published:** 2025-06-13

**Authors:** Xiaomei Zhang, Lili Huo, Ning Yuan, Zhixin Wang, Xingyun Zhu, Dan Zhao, Jianbin Sun

**Affiliations:** 1 Department of Endocrinology, Peking University International Hospital, Beijing, China; 2 Department of Endocrinology, Beijing Jishuitan Hospital, Capital Medical University, Beijing, China; Korea University Medicine, REPUBLIC OF KOREA

## Abstract

**Objective:**

Iron is increasingly recognized to influence glucose metabolism. However, evidence about the linkage between body iron stores and the risk of developing gestational diabetes mellitus (GDM) is still inconclusive. We aimed to prospectively investigate the association of serum ferritin concentrations with GDM.

**Methods:**

We studied 847 women from Peking University International Hospital from December 2017 to March 2019. Serum ferritin concentrations were measured three times during pregnancy (gestational weeks 6–12, 24–28 and 32–34). GDM was diagnosed by a 75-g oral-glucose-tolerance test (OGTT) at 24–28 weeks’ gestation. Logistic regression analyses were carried out to determine the influence of serum ferritin at the first and second trimester on the risk of developing GDM.

**Results:**

Among 847 participants, 73 women (8.6%) developed GDM. The median (IQR) of serum ferritin concentrations were 50.6 (32.4–75.5) ng/mL at gestational weeks 6–12, 19.7 (12.0–28.4) ng/mL at gestational weeks 24–28 and 19.4 (11.4–27.2) ng/mL at gestational weeks 32–34. The median serum ferritin concentrations were all significantly higher in women with GDM than those without GDM at the first, second and third trimester. Ferritin concentrations were positively correlated with the risk of GDM; the adjusted OR (95% CI) for highest vs lowest quartile was 2.97 (1.36, 6.51) at the first trimester and 2.64 (1.26, 5.54) at the second trimester.

**Conclusions:**

Elevated serum ferritin concentrations in the first and second trimester during pregnancy are both independently associated with increased risk of GDM.

## Introduction

Gestational diabetes (GDM) is a condition in which women develop high blood glucose levels during pregnancy. The prevalence of GDM has been on the rise in China [1]. A meta-analysis published in 2019 based on studies that utilized the criteria of the International Association of Diabetes and Pregnancy Study Groups, reported a pooled prevalence of GDM in mainland China at 14.8% [2]. GDM can lead to adverse maternal, fetal, and neonatal outcomes, such as an elevated risk of pre-eclampsia, preterm birth, cesarean delivery, macrosomia [3,4] or a significantly elevated risk of the mother developing diabetes after delivery [5,6].

Iron plays a crucial role in oxygen delivery, electron transport, and enzymatic activity, making it indispensable for all cell functions. During pregnancy, the demand for iron dramatically increases due to the expansion of maternal blood volume and the growth and development of the fetus [7]. Consequently, routine iron supplementation throughout pregnancy is a widely adopted global strategy to lessen the risk of maternal anemia and improve pregnancy outcomes, especially for women who are already anemic [8–10]. However, iron overload can be detrimental, as it can induce oxidative stress and directly deposit in the liver and pancreatic β-cells, leading to insulin resistance and a subsequently decrease in insulin secretion [11–13]. Emerging epidemiological studies have indicated that higher serum ferritin levels, a commonly indicator of body iron stores, in the early stages of pregnancy are associated with an increased risk of GDM [14–22]. But some previous studies were case-control studies [15,18,21]. Besides, most studies relied on a single ferritin measurement, and few have examined these associations with longitudinal measurements of serum ferritin. Additionally, the potential confounding effect of subclinical inflammation and its connection with GDM has been less considered in this context [14,16,17,19–21], given that ferritin is also an acute phase reactant and increases in various chronic diseases and inflammation conditions.

Therefore, the aim of this study was to prospectively explore levels of serum ferritin throughout pregnancy and their relationships with the subsequent risk of developing GDM, while taking into account potential confounders, including systemic inflammation as assessed by C-reactive protein (CRP).

## Methods

### Study population

This study employed a prospective cohort design. Pregnant women who routinely attended the obstetrics clinic of Peking University International Hospital for prenatal examinations between 1 December 2017 and 31 March 2019 were enrolled. Inclusion criteria were as follows: pregnant women aged 19–45 years, singleton pregnancy without diabetes, hypertension or other major chronic diseases at recruitment and planned to complete regular obstetric examinations and the final delivery at the current hospital. Women who provided blood samples for ferritin measurements during the first and second trimesters and underwent the 75-g oral glucose tolerance test (OGTT) screening during the second trimester were eligible. At the time of enrollment, we additionally ruled out acute infectious diseases such as urinary tract infection, acute respiratory tract infection and acute vaginitis. Of the 1004 women who fulfilled these criteria, 145 had a history of GDM, 7 had a low level of hemoglobin (<100g/l), and 5 had a high level of hemoglobin (≥160g/l) at gestational weeks 6–12. Totally, this analysis comprised 847 pregnant women. Self-reported prepregnancy weight and height (measured at enrolment) were used to compute prepregnancy body mass index (BMI).

The study protocol was approved by the Bioethics Committee of Peking University International Hospital (2017–021 BMR). The written informed consent has been signed by each participant.

### Ascertainment of GDM

The assessment of GDM was performed using a 75-g OGTT between 24 and 28 weeks of pregnancy. The participants underwent a minimum of three days of an unrestricted diet and unlimited physical activity, and then the 75-g OGTT was administered, following an overnight fast of at least eight hours. Plasma glucose was tested before the 75 g glucose load, as well as one and two hours later. When any of the three values was above the glucose threshold (fasting ≥ 5.1 mmol/L; 1-h ≥ 10.0 mmol/L or 2-h ≥ 8.5 mmol/L), the diagnosis of GDM was made [4].

### Biomarker measurement

Blood samples were taken by certified nurses at 3 time points: 6–12 weeks, 24–28 weeks and 32–34 weeks of gestation. Serum ferritin concentrations were measured using an electrochemiluminescence assay (Abbott i2000). The inter-assay CV was 11.0%. Plasma CRP was measured using a highly sensitive latex-particle-enhanced immunoturbidimetric assay kit on the Roche Modular P chemistry analyser. The inter- and intra-assay coefficients of variation were less than 13%.

### Statistical analysis

All data analyses were carried out using SPSS version 23.0 (SPSS Inc., Chicago, IL, USA). Data were presented as the median (25th, 75th quartile) and mean ± standard deviation for continuous variables, and as n (%) for categorical variables. Quantitative variables between GDM and non-GDM pregnant women were compared using Student’s t-test for unpaired samples. Categorical variables were compared by the χ^2^-test, and a value of P < 0.05 was considered to be statistically significant.

Due to the skewed distribution of the serum ferritin levels, quartiles of the serum ferritin at the first and second trimesters of pregnancy were used in the subsequent analyses. Baseline characteristics were compared according to quartiles of serum ferritin concentrations during the first trimester of pregnancy by ANOVA. Using the lowest quartile as the reference group, logistic regression analyses were performed to ascertin the impact of elevated serum ferritin at the first and second trimester on the risk of developing GDM. The odds ratios (ORs), adjusted ORs and their 95% CIs from the logistic regression were computed, along with the P value for the trend. Multivariable models were adjusted for potential confounding variables such as maternal age, prepregnancy BMI, haemoglobin, fasting plasma glucose and CRP.

## Results

**Fig 1:**
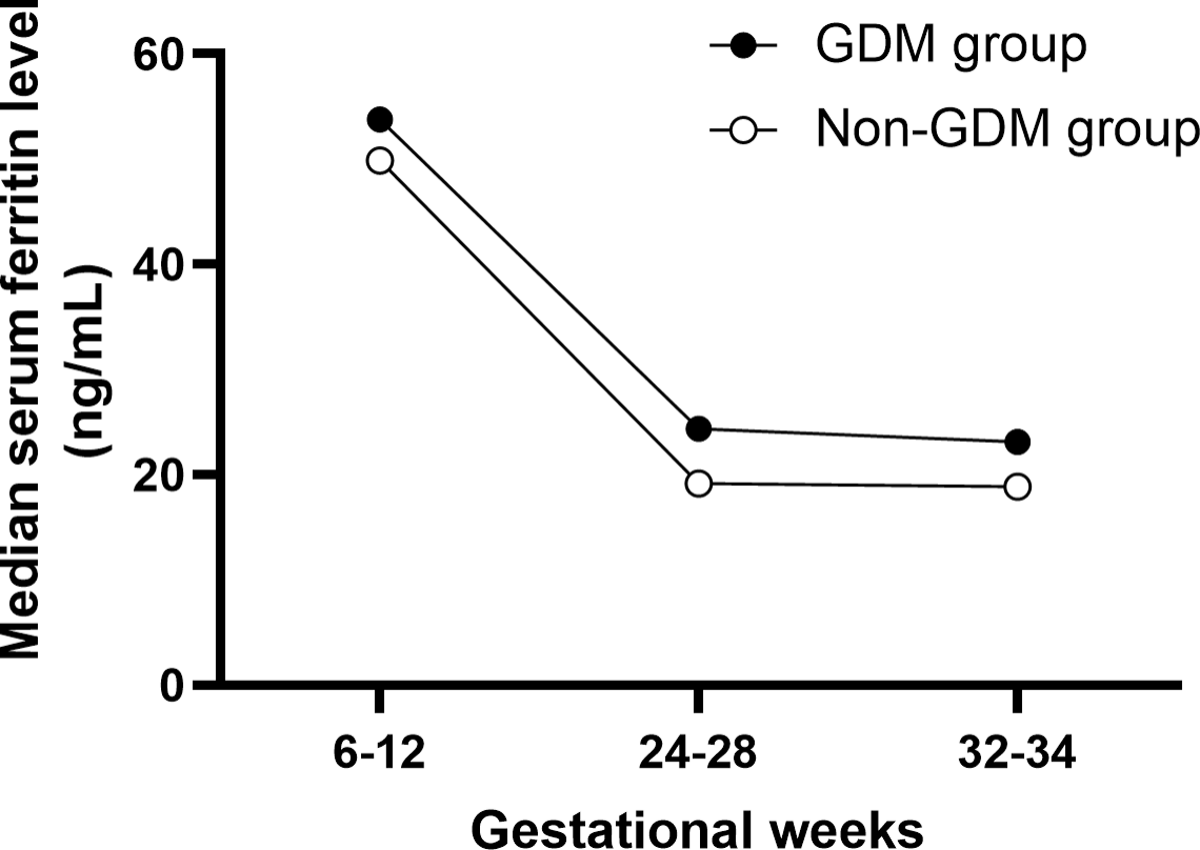
Median serum ferritin levels across trimesters among women with GDM and non-GDM.

The present study comprised 847 participants in the final analyses. Of these, 73 women (8.6%) were diagnosed with GDM. Compared with women without GDM, women with GDM were older and more likely to have a higher prepregnancy BMI, a higher fasting plasma glucose and CRP at gestational weeks 6–12. The GDM group had significantly lower prevalence of primigravida and primiparity. The two groups did not differ in haemoglobin at gestational weeks 6–12. The median (IQR) of serum ferritin concentrations in our study population were 50.6 (32.4–75.5) ng/mL at gestational weeks 6–12, 19.7 (12.0–28.4) ng/mL at gestational weeks 24–28 and 19.4 (11.4–27.2) ng/mL at gestational weeks 32–34 (Table 1). The median serum ferritin concentrations were higher in women with GDM than those without GDM at gestational weeks 6–12, which decreased during the second and third trimester, irrespective of GDM or not. The significantly higher levels of serum ferritin in women with GDM were observed across three trimesters, and the difference of serum ferritin between these two groups seemed to be greater at gestational weeks 24–28 and 32–34 as compared to gestational weeks 6–12 (Fig 1).

**Table 1:** Comparison of demographic, clinical and metabolic characteristics between women with GDM and those with non-GDM.

Characteristics	GDM group	Non-GDM group	*p* value
Number (%)	73 (8.6)	774 (91.4)	
Age (years)	32.5 ± 3.9	30.4 ± 3.5	<0.001
Pre-pregnancy BMI (Kg/m^2^)	22.9 ± 3.7	21.7 ± 3.0	0.012
Gravidity			
Primigravid	26(35.6)	390 (50.4)	0.020
Multigravid	47(64.4)	384 (49.6)	
Parity			
Primiparous	36 (49.3)	524 (67.6)	0.003
Multiparous	37 (50.7)	250 (32.3)	
Plasma biomarkers at gestational weeks 6–12			
Plasma ferritin (ng/mL)	53.8 (41.1, 95.0)	49.9 (31.6, 74.3)	0.019
Haemoglobin (g/L)	128.4 ± 9.3	129.6 ± 9.3	0.298
FPG (mmol/L)	5.0 ± 0.4	4.9 ± 0.5	0.016
CRP (mg/L)	1.59 (0.94, 3.25)	1.15 (0.55, 2.15)	0.001
Plasma biomarkers at gestational weeks 24–28			
OGTT-FPG (mmol/L)	5.0 ± 0.7	4.5 ± 0.3	<0.001
OGTT-1hPG (mmol/L)	9.3 ± 1.7	7.3 ± 1.3	<0.001
OGTT-2hPG (mmol/L)	7.9 ± 1.8	6.6 ± 1.0	<0.001
Plasma ferritin (ng/mL)	24.4 (12.7, 35.0)	19.2 (12.0, 27.8)	0.004
Plasma ferritin (ng/mL) at gestational weeks 32–34	23.1 (16.2, 31.6)	18.9 (11.4, 26.4)	0.007

We divided participants into four groups based on their serum ferritin quartiles at gestational weeks 6–12 (Q1: ≤ 32.4 ng/mL, N = 212; Q2: 32.5–50.6 ng/mL, N = 226; Q3: 50.7–75.6 ng/mL, N = 198; and Q4: ≥ 75.7 ng/mL, N = 211), and their characteristics are displayed in Table 2. No significant difference in terms of maternal age, prepregnancy BMI, fasting plasma glucose and CRP was found among the four quartiles. Women in the lowest quartile of serum ferritin at gestational weeks 6–12 had a significantly lower level of haemoglobin.

**Table 2:** Basic characteristics according to serum ferritin quartiles at gestational weeks 6-12.

Variables	Total	Serum ferritin quartiles (ng/mL)				
		Quartile 1 (≤ 32.4)	Quartile 2 (32.5-50.6)	Quartile 3 (50.7-75.6)	Quartile 4 (≥75.7)	*p* value
n	847	212	226	198	211	
Median of serum ferritin (ng/mL)	50.6	21.7	42.5	63.0	99.3	
Maternal age (years)	30.6 ± 3.6	30.7 ± 3.7	31.0 ± 3.8	30.3 ± 3.5	30.3 ± 3.4	0.184
Pre-pregnancy BMI (Kg/m^2^)	21.8 ± 3.1	22.1 ± 3.6	21.6 ± 2.8	21.8 ± 3.0	21.7 ± 3.0	0.405
Haemoglobin (g/L)	129.5 ± 9.3	127.6 ± 9.5	130.1 ± 9.5	130.5 ± 9.2	129.5 ± 8.9	0.008
FPG (mmol/L)	4.9 ± 0.5	4.9 ± 0.8	4.9 ± 0.4	4.9 ± 0.3	4.9 ± 0.4	0.665
CRP (mg/L)	1.20 (0.59, 2.27)	1.11 (0.55, 1.97)	1.21 (0.54, 2.64)	1.11 (0.56, 2.12)	1.30 (0.70, 2.27)	0.567

Serum ferritin concentrations were significantly and positively correlated with the risk of GDM (Table 3). After adjusting major risk factors of GDM, including maternal age and prepregnancy BMI, the ORs comparing the highest with the lowest quintiles of ferritin at gestational weeks 6–12 demonstrated a nearly three-fold increased odds of GDM with P-linear trend = 0.00 across quartiles. The association was not attenuated and remained significant after further adjustment for haemoglobin, fasting plasma glucose and CRP at gestational weeks 6–12. The association between serum ferritin at gestational weeks 24–28 and GDM is also detailed in Table 3. Comparing the highest with the lowest quartile, the adjusted OR associated with the risk of GDM was 2.64 (1.26, 5.54) for serum ferritin at gestational weeks 24–28.

**Table 3:** Association of plasma ferritin quartiles with risk of GDM.

Variable	GDM, n (%)	OR (95% CI)	OR (95% CI) ^a^	OR (95% CI) ^b^	OR (95% CI) ^c^
Plasma ferritin at gestational weeks 6–12					
Quartile 1 (≤ 32.4):	12 (5.7)	1.0 (Ref)	1.0 (Ref)	1.0 (Ref)	1.0 (Ref)
Quartile 2 (32.5–50.6):	15 (6.6)	1.62 (0.77, 3.40)	1.69 (0.79, 3.61)	1.87(0.85, 3.88)	1.83 (0.81, 4.12)
Quartile 3 (50.7–75.6):	20 (10.1)	1.37 (0.62, 3.00)	1.52 (0.68, 3.40)	1.60 (0.68, 3.64)	1.57 (0.67, 3.70)
Quartile 4 (≥75.7):	26 (12.3)	2.34 (1.15, 4.78)	2.72 (1.31, 5.66)	2.90 (1.36, 6.18)	2.97 (1.36, 6.51)
Plasma ferritin at gestational weeks 24–28					
Quartile 1:	13 (6.5)	1.0 (Ref)	1.0 (Ref)		1.0 (Ref)
Quartile 2:	8 (4.0)	0.59 (0.24, 1.46)	0.54 (0.21, 1.34)	0.59 (0.23, 1.49)	0.61 (0.24, 1.57)
Quartile 3:	22 (11.1)	1.80 (0.88, 3.68)	1.69 (0.81, 3.51)	1.79 (0.85, 3.77)	1.72 (0.79, 3.73)
Quartile 4:	30 (14.9)	2.52 (1.28, 5.00)	2.55 (1.26, 5.13)	2.58 (1.26, 5.28)	2.64 (1.26, 5.54)

^a^Adjusted for maternal age and pre-pregnancy BMI.

^b^Adjusted for maternal age and pre-pregnancy BMI, haemoglobin, fasting plasma glucose at gestational weeks 6–12.

^c^Adjusted for maternal age, pre-pregnancy BMI, haemoglobin, fasting plasma glucose and CRP at gestational weeks 6–12.

## Discussion

In this prospective, longitudinal study of pregnant women without prepregnancy chronic illnesses, we have demonstrated associations between higher serum ferritin during early and middle pregnancy and the risk of GDM, even after controlling for plasma CRP levels, prepregnancy BMI and other main GDM risk factors. Furthermore, the magnitude of associations appeared similar between the first and the second trimester.

The median serum ferritin in our population was 50.6ug/L at the first trimester, which was comparable to the median level of 59.4 ug/L found in another recent Chinese study [17]. In addition, we investigated the longitudinal trajectory of serum ferritin levels acorss the whole gestational period, and found that ferritin levels declined by more than half at the second trimester compared to the first trimester and then tended to be stable at the third trimester. China guideline on the management of iron deficiency and iron deficiency anemia in pregnancy recommends a serum ferritin level of < 20 ug/L as an indicator of iron deficiency and oral iron supplementation if serum ferritin ≤30 ug/L [9]. The prevalence of iron deficiency in our population was 10.2% in early pregnancy, 54.2% in middle pregnancy and 55.1% in late pregnancy using a serum ferritin level < 20ug/L, suggesting inadequate adherence to national guidance.

However, iron is regarded as a two-edged sword in biological systems since both an excess or a shortage of iron can be detrimental. In this prospective cohort study of pregnant women, the ORs comparing the highest with the lowest quartile of ferritin at gestational weeks 8 demonstrated a nearly 3-fold increased odds of GDM after adjustment for main risk factors of GDM, including maternal age, prepregnancy BMI and CRP. Similar findings were reported in several earlier studies. In 2006, the Camden Study reported women in the highest quintile of serum ferritin had a 2-fold increased risk of GDM, but the effect became non-statistically significant after additional adjustment for prepregnancy BMI or CRP [22], which suggested that low-grade inflammation or obesity may act as a partial mediating factor for the increased risk of GDM associated with an elevated serum ferritin level. There may be potential concerns as the Camden Study seemed not to rule out women with known inflammatory diseases at entry to care and stored blood samples were used for measurements. Cheng’s study revealed that serum ferritin during the first trimester significantly elevated the incidence of GDM after adjusting several established risk factors including prepregnancy BMI and inflammation in a Chinese urban population [17]. However, the marker of inflammation used was plasma white blood cell count rather than CRP. A case–control study published in 2016 using data from the Danish National Birth Cohort demonstrated that women in the highest quintile of plasma ferritin measured at 6–12 gestational weeks had a statistically significant and > 2-fold increased risk of developing GDM after controlling for main risk factors of GDM, including CRP and prepregnancy BMI, compared with the lowest quintile [18]. Another case-control study published in 2017 measured serum ferritin levels at 10–14 and 15–26 weeks of gestation, and found that ferritin levels were positively associated with GDM risk after adjusting confounders including CRP and prepregnancy BMI; the aOR (95% CI) for highest vs lowest quartile was 2.43 (1.12, 5.28) at weeks 10–14 and 3.95 (1.38, 11.30) at weeks 15–26, which was consistent with our findings. However, some studies reported opposite results. Zeni et al. found that ferritin concentrations positively correlated to 2-h OGTT glucose value, but were not related to the incidence rate of GDM, possibly in reason to the small sample size of their study [23]. Chan et al.’s study was a randomized placebo-controlled trial to explore whether iron supplement from early pregnancy would increase the risk of GDM and found that no significant difference in the incidence of GDM between the iron supplementation and placebo groups at 28 weeks or 36 weeks despite the significantly higher serum ferritin levels in the iron supplementation groups [24]. But there was no significant difference in the baseline ferritin (around gestational week 11) between the two groups.

Recently, some studies investigated the dose-response pattern between ferritin levels and GDM, and yielded inconsistent results. Zhang et al. reported continuous positive association between plasma ferritin and the risk of GDM [14], while chen et al. reported a U-shaped association [20]. Chen et al.’s study utilized 10–90th percentile of serum ferritin as the reference group and found that women with serum ferritin below the 10th percentile exhibited a higher risk of GDM in comparison to the reference group. The median ferritin in the < 10th percentile group was 15.6 (8.66, 19.7) ng/mL, which was much lower than that in the bottom quartile group of our study. Zhang et al.’s study employed bottom quartile of serum ferritin as the reference group with a median level of 21.7 (16.0, 27.7) ng/mL, which was comparable with our study.

Several potential mechanisms may be implicated in the role of iron overload in driving glucose metabolism abnormalities. Iron induces the production of reactive oxygen species and lipid peroxidation [12], which can trigger ferroptosis [25]. Pancreatic beta-cells are susceptible to ferroptotic cell death with intrinsically lower expression and activity of antioxidant enzymes [26,27]. The lipid peroxidation will reduce glucose utilization in muscle tissue and enhance gluconeogenesis, leading to insulin resistance [11,13]. In addition, iron may also have an impact on insulin action by modulating adipocyte differentiation and affecting the release of adipokines at adipose tissue [11,13].

The current study is one of very few prospective, longitudinal studies with a relatively large sample size to examine the role of serum ferritin in the process of GDM development. Serum ferritin levels were measured longitudinally throughout each of the three trimesters of pregnancy, which allows to observe the trajectory of serum ferritin levels during pregnancy. The measurement of serum ferritin levels at the first trimester is well before the diagnosis of GDM, which is critical to rule out the possibility of inverse causation. However, several limitations need to be considered when interpreting the results. First, there could be residual confounders due to the availability of information. While we have taken confounders such as maternal age, CRP and pre-BMI into account in our analyses, risk factors for GDM such as socioeconomic status, family history of diabetes, physical activity or weight gain during pregnancy were left out. Second, we only focused on the relationship of ferritin levels to GDM risk. To better elucidate the role of iron in the development of GDM, investigations on a variety of iron markers in combination with dietary and supplemental iron are warranted. Third, the medical and family history of hereditary iron metabolism disorders was not screened in our study population. Participants with hemoglobin < 100g/l or ≥ 160g/l at the time of enrollment were ruled out and hereditary iron metabolism disorders are relatively rare conditions, therefore, we do not think that the major findings will be affected by this potential bias. Lastly, the population of China is heterogeneous, and our study cohort was from one single center, which may limit the generalizability of the findings.

In conclusion, findings from this study indicate that elevated serum ferritin concentrations during the first and second trimester of pregnancy are both independently linked with an increased risk of developing GDM. Since iron deficiency remains a major concern for pregnant women, further research with comprehensive evaluations of iron status and iron intake is required to explore individually tailored use of iron supplementation and improve outcomes for pregnant women.

## Supporting information

S1 File STROBEchecklist. (DOCX)
